# Headache service quality evaluation: implementation of quality indicators in primary care in Europe

**DOI:** 10.1186/s10194-021-01236-4

**Published:** 2021-04-28

**Authors:** B. Lenz, Z. Katsarava, R. Gil-Gouveia, G. Karelis, B. Kaynarkaya, L. Meksa, E. Oliveira, F. Palavra, I. Rosendo, M. Sahin, B. Silva, D. Uludüz, Y. Z. Ural, I. Varsberga-Apsite, S. T. Zengin, L. Zvaune, T. J. Steiner, on behalf of European Headache Federation and Lifting The Burden: the Global Campaign against Headache

**Affiliations:** 1 Department of Neurology, Evangelical Hospital Unna, Unna, Germany; 2 Department of Neurology, Bundeswehr Central Hospital Koblenz, Koblenz, Germany; 3 Department of Neurology, University of Duisburg-Essen, Essen, Germany; 4 EVEX Medical Corporation, Tbilisi, Republic of Georgia; 5 IM Sechenov First Moscow State Medical University (Sechenov University), Moscow, Russian Federation; 6 Hospital da Luz Headache Center, Lisbon, Portugal; 7 Riga East Clinical University Hospital, Neurology and Neurosurgery Department, Headache Unit, Riga, Latvia; 8 Kagıthane Yahya Kemal ASM, Istanbul, Turkey; 9 Faculty of Medicine, University of Coimbra, Coimbra, Portugal; 10 Centre for Child Development – Neuropediatrics Unit, Hospital Pediátrico, Centro Hospitalar e Universitário de Coimbra, Coimbra, Portugal; 11 Family Health Unit “Coimbra Centro”, Coimbra, Portugal; 12 Kartal 10 Nolu ASM Istanbul, Istanbul, Turkey; 13 Family Health Unit “Pulsar”, Coimbra, Portugal; 14 Neurology Department, Istanbul University Cerrahpasa School of Medicine, Istanbul, Turkey; 15 Esenler Havaalanı ASM, Istanbul, Turkey; 16 Bagcılar Yıldıztepe ASM, Istanbul, Turkey; 17 Norwegian University of Science and Technology, Trondheim, Norway; 18 Division of Neuroscience, Imperial College London, London, UK

**Keywords:** Headache disorders, Headache care, Primary care, Service quality evaluation, Quality indicators, Global campaign against headache

## Abstract

**Background:**

*Lifting The Burden* (LTB) and European Headache Federation (EHF) have developed a set of headache service quality indicators, successfully tested in specialist headache centres. Their intended application includes all levels of care. Here we assess their implementation in primary care.

**Methods:**

We included 28 primary-care clinics in Germany (4), Turkey (4), Latvia (5) and Portugal (15). To implement the indicators, we interviewed 111 doctors, 92 nurses and medical assistants, 70 secretaries, 27 service managers and 493 patients, using the questionnaires developed by LTB and EHF. In addition, we evaluated 675 patients’ records. Enquiries were in nine domains: diagnosis, individualized management, referral pathways, patient education and reassurance, convenience and comfort, patient satisfaction, equity and efficiency of headache care, outcome assessment and safety.

**Results:**

The principal finding was that Implementation proved feasible and practical in primary care. In the process, we identified significant quality deficits. Almost everywhere, histories of headache, especially temporal profiles, were captured and/or assessed inaccurately. A substantial proportion (20%) of patients received non-specific ICD codes such as R51 (“headache”) rather than specific headache diagnoses. Headache-related disability and quality of life were not part of routine clinical enquiry. Headache diaries and calendars were not in use. Waiting times were long (e.g., about 60 min in Germany). Nevertheless, most patients (> 85%) expressed satisfaction with their care. Almost all the participating clinics provided equitable and easy access to treatment, and follow-up for most headache patients, without unnecessary barriers.

**Conclusions:**

The study demonstrated that headache service quality indicators can be used in primary care, proving both practical and fit for purpose. It also uncovered quality deficits leading to suboptimal treatment, often due to a lack of knowledge among the general practitioners. There were failures of process also. These findings signal the need for additional training in headache diagnosis and management in primary care, where most headache patients are necessarily treated. More generally, they underline the importance of headache service quality evaluation in primary care, not only to identify-quality failings but also to guide improvements.

This study also demonstrated that patients’ satisfaction is not, on its own, a good indicator of service quality.

**Supplementary Information:**

The online version contains supplementary material available at 10.1186/s10194-021-01236-4.

## Background

Headache disorders, although a major public-health problem, are largely treatable without need for specialist intervention, but effective care fails to reach large numbers of people worldwide [1, 2]. New headache drugs are not the answer. Although there is need for better drugs, their impact will be minimal if introduced into systems of care that cannot effectively make use of them – any more than those available already. Ten years ago, better education of medical professionals was proclaimed as the most-needed prerequisite for improving headache care [1]. In primary care management of headache, education does indeed improve practice [3].

But there is need to take a much more expansive view: the concept of *service quality* must take centre-stage in headache care, wherever it is delivered [4]. Service quality has broader meaning than quality of care. This and many other factors contribute to service quality, just as many causes lead to its failure.

Some years ago, as a key project within the Global Campaign against Headache [5], *Lifting The Burden* (LTB) and the European Headache Federation (EHF) assembled a group of experts to define *quality* in the context of headache care. “Good quality headache care achieves accurate diagnosis and individualized management, has appropriate referral pathways, educates patients about their headaches and their management, is convenient and comfortable, satisfies patients, is efficient and equitable, assesses outcomes and is safe” [6]. The experts analyzed and evaluated putative quality indicators, requiring that they reflect patients’ and public-health priorities and be applicable in different settings and cultures as well as to all types of headache. A total of 30 indicators were eventually assigned to nine verifiable quality domains (Table 1) [6].

**Table 1:** The nine domains of quality in a headache service (adapted from [6])

Domain A	Diagnostic accuracy: appropriate enquiry; diagnoses according to IHS criteria, documented during the first visit, reviewed during follow-up and supported by diagnostic diaries
Domain B	Individualized management: waiting time tailored to urgency; adequate time allocation; evidence-based treatment plans reflecting diagnosis and disability, with follow-up
Domain C	Availability and utilization of urgent and specialist referral pathways
Domain D	Patient education and reassurance
Domain E	Convenient, clean, comfortable and welcoming service
Domain F	Patient satisfaction
Domain G	Equitable access to care, efficiency of care, cost-controls and avoidance of wastage
Domain H	Outcome measures of symptoms, disability and quality of life
Domain I	Safety of care

In a series of evaluations, these indicators were first implemented in a pilot study in two highly specialized headache centres (at the University Hospital Essen, Germany, and the Hospital de Luz in Lisbon, Portugal) using the questionnaires developed for doctors, other health-care providers (HCPs), service managers, secretaries or administrators and patients [7]. A definitive study followed in 14 specialist-care centres, extending this evaluation across Europe [8]. Both studies found the quality indicators to be practical in specialist care and fit for purpose at this level: treatment deficits were identified and eliminated [8].

The essential next step in evaluation is to take the process into non-specialist care, including primary care, where the large majority of headache patients must be treated [4]. This study has this objective, continuing the service quality evaluation (SQE) collaborative project between LTB and EHF [5]. Its primary purpose is to assess the applicability and practical operation of the quality indicators in primary care. Its secondary purpose is to assess the quality of headache management currently in primary care in Europe, identifying deficits and providing guidance for improvement.

## Methods

### Ethics

Approvals were obtained in each country in accordance with local regulations (some did not require ethics approval for studies with the primary purpose of service quality improvement). Informed consent was obtained from all study participants, regardless of whether or not ethics approval had been required.

### Study settings and participants

During 2019, a total of 53 primary-care practices from four European countries were invited to participate (10 from Germany, five from Turkey, five from Latvia and 33 from Portugal), identified through personal contacts and selected to represent, as far as possible, the geographic distinctions of the four countries. Of these, 25 (47%) declined for various reasons (commonly lack of time or interest). The study was therefore conducted in 28 (four in Germany, four in Turkey, five in Latvia and 15 in Portugal).

In each clinic we interviewed doctors (general practitioners [GPs]), other HCPs (practice nurses and/or medical assistants), service managers, secretaries and/or administrators and patients, and evaluated consecutive patients’ records.

### Study instruments

The data were collected prospectively under the supervision of the local principal investigator using the prescribed SQE questionnaires for each group of interviewees and for extraction of data from patients’ records. The questionnaires were adapted to the primary-care setting (essentially with regard to referral pathways) and translated into the local language(s) according to LTB’s translation protocols [9]. The questionnaires, in their original English, are attached in Additional Files 1, 2, 3, 4 and 5.

### Procedure

In each clinic, staff were first informed of the aims and nature of the study and of the types of data to be collected, then interviewed by the local principal investigator using the appropriate questionnaires (Additional files 1, 2, 3 and 4).

All adult patients attending during the study period with the symptom “headache” were asked for interview, which proceeded with their consent. These interviews were semi-structured (Additional file 5), and conducted either by the local investigator or by GPs or other HCPs from the service trained for the purpose by the local investigator. In addition, local investigators reviewed the records of these and other randomly selected patients, extracting relevant information (Additional file 6).

Further information on the quality indicators for headache care services and their application can be found in Additional File 7.

### Data management and analysis

Data were entered locally and anonymously into spreadsheets provided, and in this form transferred to the data collection centre (Clinic for Neurology, Geriatric Medicine and Neurorehabilitation in the Evangelical Hospital Unna), where they were merged and analyzed descriptively. Demographic and clinical data were provided as numerical values and summarized as percentages or mean values with standard deviations (SDs). Analyses included comparison of findings with those from 14 previously analyzed specialist-care centres [8].

No hypotheses were formulated.

Analyses and comparisons were completed in Microsoft Excel® 2016.

## Results

We interviewed 111 doctors, 92 nurses and medical assistants, 70 secretaries or administrators, 27 service managers and 493 patients (122 in Germany, 125 in Turkey, 156 in Latvia and 90 in Portugal), and evaluated 675 patients’ records (150 in Germany, 125 in Turkey, 250 in Latvia and 150 in Portugal) (Table 2). The participating practices had similar structures but differed in size and staffing, with one to 14 doctors, one to ten other HCPs, up to two practice managers and one to seven administrative staff.

**Table 2:** Characteristics of participating primary-care practices

Practice characteristics		Germany	Turkey	Latvia	Portugal	Total
**Practices**	n	4	4	5	15	28
**Staff**	Doctors (GPs), n	9	4	5	93	111
	Medical assistants, n	15	0	3	0	18
	Nurses, n	0	4	2	68	74
	Managers, n	6	4	5	12	27
	Secretaries / administrators, n	12	4	5	49	70
**Patients**	n	122	125	156	90	493
	Female (%)	78	75	72	81	77
	Mean age (yr ± SD)	44.5 ± 15.7	39.7 ± 14.2	39.9 ± 16.5	45.8 ± 17.8	
	Mean duration of headache (yr ± SD)	17.8 ± 14.5	6.9 ± 7.5	2.3 ± 4.1	1.9 ± 4.6	
**Patients’ records**	n	150	125	250	150	675
**Diagnoses**	Migraine unspecified (G43.9, G43.8), n	54	47	28	24	153
	Episodic migraine without aura (G43.0), n	12	0	1	0	13
	Episodic migraine with aura (G43.1), n	6	1	4	0	11
	Chronic migraine (G43.3), n	2	7	0	0	9
	Tension type headache (G44.2), n	3	47	23	20	93
	Cluster headache (G44.0), n	4	1	3	0	8
	Other primary or secondary headache disorders (summarized under G44.8), n	1	6	52	0	59
	Medication-overuse headache (G44.4), n	0	0	0	0	0
	Mononeuritis / Occipital neuralgia (G58.8)	9	0	0	0	9
	Headache unspecified (R51), n	30	7	35	27	99
	Others, n	1	9	10	19	39

Patients were aged 18–86 years and mostly (77%) female (Table 2). Duration of headache as the presenting complaint ranged from 1 day to 50 years, with country means varying widely from 1.9 ± 4.6 years in Portugal to 17.8 ± 14.5 years in Germany (Table 2).

Local investigators were able to collect required data quickly and efficiently from the questionnaires in all countries. All interviewed staff reported that these were easy to use and understand, and not unduly time consuming. The few comprehension difficulties (for example, “What is an outcome measure that is based on self-reported disability burden?” or “What is meant by a ‘formal triage system’?”) were due to lack of knowledge. These uncertainties were reflected in the limited use of outcome assessment instruments (see below).

Findings with regard to each individual quality indicator in the nine quality domains are presented by country in Table 3. The following is a narrative summary.

**Table 3:** **Responses to questionnaire enquiries** (% of positive answers, except where specified)

Domain and indicator	Information source(s)	Enquiry	Germany	Turkey	Latvia	Portugal	Average
A1a	Patients’ records	Is duration of presenting complaint recorded in patient’s record?	43	98	20	51	47
A1b	Patients’ records	Are frequency or days/month of symptoms recorded in patient’s record?	25	88	8	30	32
A2a	Patients’ records	Is diagnosis recorded in patient’s record?	100	65	82	missing	83
A2b	Patients’ records	Does diagnostic record use ICHD terminology?	72	46	53	41	53
A3	Patients’ records	Is working diagnosis at first visit recorded in patient’s record?	59	62	86	72	72
A4	Patients’ records	Is definitive diagnosis recorded in patient’s record or, if not, has an appointment for review been given?	68	46	77	62	66
A5	Doctors	Is it routine practice in your headache service to review a patient’s diagnosis during follow-up?	78	75	100	95	93
A6	Doctors, manager	Are diagnostic diaries available in your headache service?	0	25	30	26	23
B1a	Doctors, manager, secretary	Does a formal triage system exist in your headache service?	52	8	47	17	23
B1b	Doctors, manager, secretary	(If yes) Is your triage system designed to pick out potentially urgent cases for early appointments?	100	100	100	73	85
B2a	Patients	Time per visit in minutes (mean ± SD)	17 ± 8	8 ± 4	17 ± 7	24 ± 12	16 ± 9
B2b	Patients	Satisfaction with time per visit (“about right”)	84	83	71	99	82
B2c	Doctors, other HCPs	Are you satisfied that sufficient time is allocated to each patient’s visit to enable a good management?	63	50	50	31	36
B4	Doctors, other HCPs, manager	Does an access route to psychological therapies exist in your headache service?	67	33	33	67	63
B5	Doctors, other HCPs, manager	Is an instrument for disability assessment available in your headache service?	7	8	33	21	19
B6a	Doctors, other HCPs, manager	Does your headache service allow follow-up of every patient who needs it?	97	67	100	missing	91
B6b	Doctors, other HCPs, manager	Is a follow up diary or calendar available in your headache service?	23	25	40	24	25
C1	Doctors, manager, secretary	Does a referral pathway exist?	85	0	33	90	80
C2	Doctors, manager, secretary	(If yes) Does this pathway permit, and respond to, urgent referral when needed?	83	0	100	83	84
D1a	Doctors, other HCPs, manager	Are information leaflets available?	27	0	60	14	18
D1b	Patients	Doctor provides patient with information	48	81	96	99	81
D1c	Patients	(If yes) Information given understandable?	100	97	92	99	96
D1d	Patients	(If yes) Amount of information about right	83	88	67	94	81
D2	Patients	Patients were given reassurance	91	82	86	97	88
E1a	Patients	Service environment clean and comfortable	98	82	96	98	93
E1b	Doctors, other HCPs	Service environment clean and comfortable	88	75	90	43	52
E2	Patients	Satisfaction with welcome	99	96	90	100	96
E3a	Patients	How long were you kept waiting to see the doctor (in minutes)? (mean ± SD)	57 ± 53	6 ± 7	15 ± 15	24 ± 28	24 ± 36
E3b	Doctors, other HCPs	Satisfaction with waiting time	67	100	100	85	84
E3c	Patients	Satisfaction with waiting time					
		reasonable	60	75	79	83	74
		too long	23	17	16	15	18
		much too long	17	8	5	2	8
F1	Patients	Satisfaction with overall management					
		very good	38	9	9	26	19
		good	39	36	40	28	37
		adequate	15	46	37	45	35
		poor	7	7	13	1	8
		very poor	1	2	1	0	1
G1	Manager	Protocol to limit wastage exists	33	25	20	42	33
G2	Manager	Record of input costs exists	83	0	0	58	44
G3	Doctors, other HCPs, manager	Policy to ensure equal access exists	100	67	93	81	83
H1	Doctors, other HCPs, manager	Outcome measures include Headache Under-Response to Treatment questionnaire (HURT) or similar	10	25	13	5	7
H2	Doctors, other HCPs, manager	Outcome measures include Headache-Attributed Lost Time index (HALT) or similar	0	8	0	3	3
H3	Doctors, other HCPs, manager	Outcome measures include World Health Organization Quality of Life questionnaire (WHOQoL) or similar	3	0	13	3	4
I1	Doctors, other HCPs, manager	Protocol for reporting serious adverse events exists	73	25	80	58	60

### Domain A: an accurate diagnosis is essential for optimal headache care

**Fig. 1:**
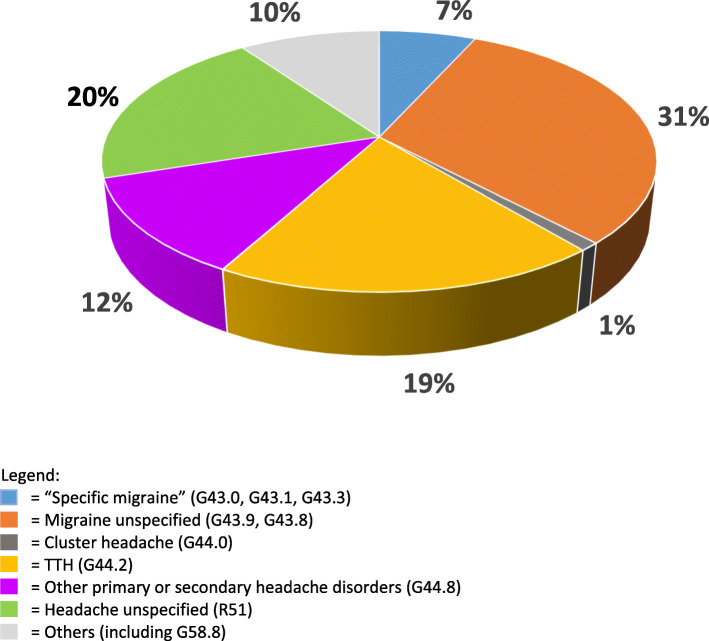
Distribution of diagnoses across practices (percent)

Temporal profiles and specific features of presenting headaches were assessed either inaccurately or not at all in almost all practices, with Turkey the exception (88%: Table 3). About two-third of clinics documented working or definitive diagnoses at first or subsequent visits and most (92%) reviewed these during later follow up. Diagnostic diaries were available in a minority (0% in Germany to 30% in Latvia), with many HCPs unaware of them and others believing they were too time-consuming. In many clinics but far from all (45% in Turkey to 72% in Germany), diagnoses were made according to the current International Classification of Headache Disorders (ICHD) criteria; nevertheless, except in Turkey (6%), a sizeable proportion of patients (22–30%) received “Headache” (unspecified) diagnoses (coded R51 in ICD-10 classification) (Fig. 1).

Among specific diagnoses, migraine (whether or not further specified) was the most common in each country, followed by tension-type headache (Table 2). Latvia stood out with a high number of “Other primary or secondary headache disorders” (Table 2). No patients in any country were diagnosed with medication-overuse headache (MOH).

### Domain B: individualized management is essential for optimal headache care

Most practices did not use a formal triage system to expedite cases from waiting lists that might need this. In Germany and Portugal, the term “formal triage system” was subject to broad interpretation across interviewee groups. For example, prior to explanation of the term by the local investigator, many secretaries or administrators, but also some physicians, stated that their “triage system” was based mainly on the “trained eye” and “experience”. Doctors argued that they were able to recognize “red flags”, and act accordingly without a formal system for the purpose.

**Fig. 2:**
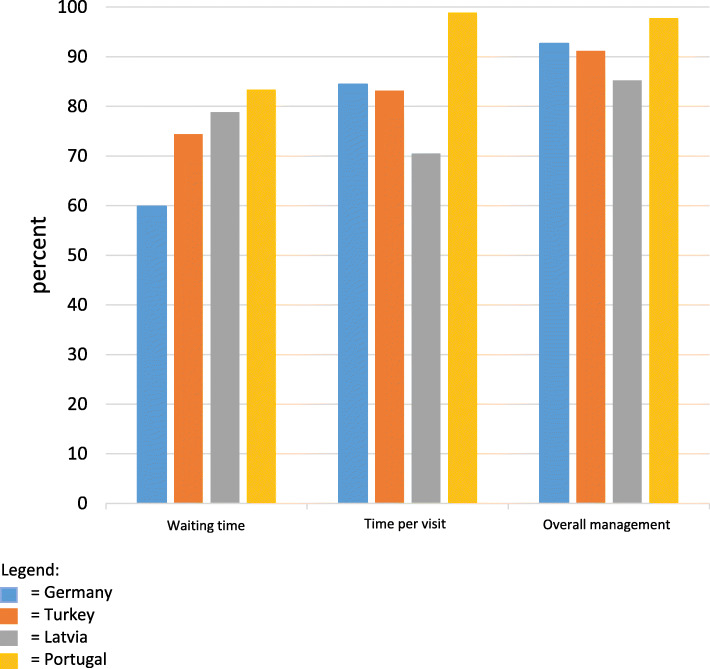
Patients’ satisfaction with three aspects of their care, by country

Patients reported mean times allocated to their visits of 16 ± 9 min. Across all countries, 82% of patients, on average, were satisfied with the time spent on them (Fig. 2). Patient satisfaction was by far the highest in Portugal (99%), where the reported average visit time was longest at 24 ± 12 min. Nearly two thirds of HCPs, however, would have preferred more time per visit.

One third of practices in Latvia, but fewer elsewhere, used an instrument for disability assessment at the time of diagnosis. However, in Germany, the recording of sick days or sick leave due to a recurring diagnosis was understood as a record of disability.

In Germany and Portugal, two thirds of practices had an access route to psychological therapies, but in Turkey and Latvia only one-third. In this context it should be mentioned that, in Germany in particular, many GPs stated that they themselves could offer psychotherapeutic or psychological treatments to their patients on account of their additional training or qualifications.

Almost all practices were able to offer their patients follow-up, when considered necessary, although few used standardized follow-up diaries or outcome assessment instruments to monitor progress.

### Domain C: appropriate referral pathways are essential for optimal headache care

In Germany and Portugal, more than 85% of HCPs indicated that appropriate onward referral pathways existed, but in Latvia only one third and in Turkey none. In Germany, the destination of referrals was largely determined by long waiting times (often several weeks to months) for outpatient specialist care, so this was not an option for time-critical referrals. Neurologists´ waiting times were sharply criticized by both patients and HCPs. Extended waiting times for elective hospital admissions were also criticized, with the result that admissions were often made via the emergency room.

### Domain D: education of patients about their headache and their management is essential for optimal headache care

As with diagnostic questionnaires and diaries, few HCPs in most countries were aware that patient information leaflets were freely available in various languages. Accordingly, except in Latvia, information leaflets were not often available. Nevertheless, the great majority of patients expressed satisfaction with the information and reassurance provided by their doctors.

### Domain E: convenience and comfort are part of the optimal headache care

More patients than HCPs considered their service environment to be clean and comfortable, most markedly in Portugal (HCPs 43%, patients 98%). Almost all patients felt welcomed. Reported waiting times varied widely (mean 24 ± 36 min), being least in Turkey (6 ± 7 min) and longest by far in Germany (57 ± 53 min); nevertheless, only a minority of patients and HCPs were dissatisfied with waiting times, and nearly 60% of German patients found them acceptable (Fig. 2).

### Domain F: achieving patient satisfaction is part of optimal headache care

Overall patient satisfaction was high in all countries, 91% rating their management adequate, good or very good (Fig. 2).

### Domain G: optimal headache care is efficient and equitable

About one-third of practices had protocols to avoid wastage of resources. Running costs were recorded in Germany (83%) and in Portugal (58%), but not in Turkey or Latvia. Most practices (83%, but only 67% in Turkey) offered equal access to their (headache) service for all patients.

### Domain H: outcome assessment is essential in optimal headache care

Outcome assessment instruments (e. g. HURT, HALT, WHOQoL) were not in use routinely in primary care. In personal conversations, it was found that the majority of HCPs in Germany were not aware of these questionnaires or other options. Headache intensity was assessed by Visual Analogue Scale (VAS). In addition in Germany, change in frequency of sick leave attributed to a long-term diagnosis served as a disability indicator and as a measure of treatment outcome.

### Domain I: optimal headache care is safe

On average nearly 60% of all general practices had formal protocols to ensure reporting of serious adverse events. Turkey (25%) was unusual in this aspect of care.

### Comparisons between specialist-care centres and primary-care practices in Europe

**Fig. 3:**
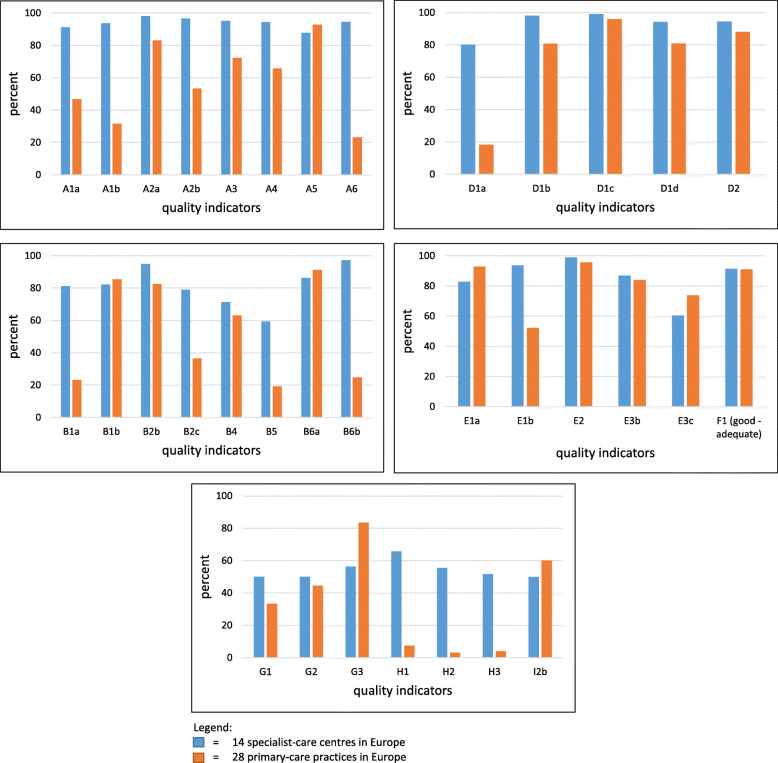
Headache service quality evaluation: comparisons between specialist and primary care in Europe in eight quality domains. A: accurate diagnosis; B: individualized management; D: education of patients; E: convenience and comfort; F: patients’ satisfaction; G: efficiency and equitability; H: outcome assessment; I: safety

We made comparisons between the 28 primary-care practices and 14 specialized-care centres, using data collected several years ago in university-based headache clinics throughout Europe [8]. Figure 3 shows these in eight quality domains (excluding referral pathways, which have different implications in primary and specialist care). Quality of headache care was inferior in primary care on almost all indicators in all domains. The most marked differences could be identified in domain A (Accurate diagnosis) and domain H (Outcome assessment), but there were notable deficits in primary care relative to specialist care in other individual quality indicators (for example in the availability of information leaflets [D1a]).

Primary care was, however, superior in providing equitable and easy access to headache care. Furthermore, the vast majority of patients were satisfied with their headache care in both primary and specialist care.

## Discussion

To our knowledge, this was the first study addressing quality of headache care in primary care. It continued and was an important extension of the evaluation process of the quality indicators for headache care developed by LTB and EHF; previous studies were conducted in specialist centres [7, 8]. Although the participating practices differed in size, staffing levels and the national health-care systems in which they were set, findings were broadly comparable between countries and common trends in practice were evident. The essential findings related to practicality of the indicators, and their fitness for purpose in primary care. Regarding the former, all interviewed staff found the questionnaires easy to use and understand, and, crucially, not unduly time consuming. As to the latter, this was evidenced in the assessments made. It was not a primary purpose of this study to assess service quality in primary care but, in doing so, the indicators demonstrated their fitness for purpose.

This was best illustrated in the comparisons between primary and specialized care: by most indicators in all quality domains, specialized headache clinics performed better than primary care. Any other finding would have cast serious doubt on the validity of the indicators. As it is, the comparisons demonstrated that the indicators can be used in different settings with the expectation of revealing quality differences. We note here that, while the indicators identify quality deficits, they do not yet determine whether or not quality is “good” in any particular setting. Future benchmarking studies are needed for this.

Among specific indicators, lack of formal triage systems was a not unexpected feature of primary care, to which, unlike specialist care, access is generally unrestricted and not subject to long and potentially harmful delays. Doctors’ assertions seemed reasonable that experience enabled triage in primary care without a formal system for the purpose. This aside, in all countries, quality deficits were uncovered, and these led inevitably to suboptimal treatment judged objectively – although this was not reflected in patients’ satisfaction ratings (we say more on this later). Most deficits were attributable to HCPs’ lack of relevant knowledge. While this might also, to an extent, be expected in non-specialist care, it needs remediation given that headache disorders are the most common single cause of consultation in primary care [10, 11]. Education of HCPs in headache was identified 10 years ago by WHO and LTB as the most pressing need, worldwide, in the pursuit of better headache care [1]. Basic but structured education of GPs has been shown to improve their practice, significantly increasing proportions of patients given specific diagnoses and treatments, both indicators of headache care quality [3, 12].

Pertaining to this, an important particular finding was that a substantial proportion of patients of these primary-care practices received non-specific ICD codes such as R51 (“headache”) rather than specific headache diagnoses. To be fair, coding was often driven by practical rather than clinical considerations, or by administrative requirements, and might not reflect diagnostic deficiencies. Nevertheless, almost everywhere, accurate diagnosis was impeded by inadequately assessed histories of headache, especially with regard to temporal profiles, unaided by simple aids such as headache diaries and calendars. It is well recognized that the history is all-important in correct headache diagnosis, itself the essential foundation of successful treatment [13], and unsurprising therefore that the Eurolight study has already demonstrated headache management to be generally suboptimal in European primary care [14]. A particularly telling finding in our study was that none of the practices gave a single diagnosis of MOH, despite that MOH is common in the general population level (prevalence ranging between 0.5% and 7.2% [15]) and that people with MOH are highly likely to seek GP consultation. Another was Latvia‘s high number of “Other primary or secondary headache disorders”, which could be summarized under ICD-10 code G44.8 and included “Headache attributed to arterial hypertension” (ICHD-3 code 10.3, *n* = 29), “Alcohol-induced headache” (ICHD-3 code 8.14, *n* = 7) and “Headache attributed to acute rhinosinusitis” (ICHD-3 code 11.5, *n* = 4). While Germany, Turkey and Portugal might have subsumed these under R51, or coded them only according to the underlying causative disorders, there is reason to question these. In particular, ICHD notes that the first of them is attributable only to severe hypertension and is uncommon [16], but in some countries a contrary belief persists.

Although it was not a primary purpose, our study emphasizes the need, in all countries studied, for wider implementation of educational programmes aimed at GPs.

Good management of headache patients begins with an explanation of their disorder and of the purpose and means of management [12]. This is time consuming. Time constraints were mentioned by doctors in all countries as a key factor impeding good management. Time can be saved, and good management supported, by offering patients information leaflets, such as those produced by LTB and freely available [13]. Every patient offered a treatment or whose treatment is changed requires follow-up in order to assess its success against expectation [13]. This, also, is time consuming, but can be aided by various outcome measurement instruments, again freely available [17, 18]. None of these were in routine use in the primary-care practices. While this was largely because of lack of awareness of them, some GPs argued that time constraints themselves made it impossible to incorporate them into everyday routine. This argument is mistaken: patients can fill in questionnaires at home, or in the waiting room, so that use of these kinds of instrument is time saving. Education can solve this, while introducing these aids into primary-care headache management is a low-cost intervention.

There were some positive findings for primary care. Most importantly, the great majority of the primary-care services provided equitable and easy access to headache care, without barriers, and were able to provide follow-up to every patient who needed it. These essential features of primary care [19, 20] distinguished it sharply from specialized care. Furthermore, patients overall were satisfied with their care: with the time allocated to them, with the care environment and with their management overall. There are three points to be made here. First, these evaluations reflect the common trust of patients in family doctors all over the world [19, 21–24], which is at the heart of good patient-doctor interaction. Second, they show the importance attributed by patients to structure and process [6, 25–28]. Third, and of particular significance in this context, is that patients’ satisfaction, while essential as a quality indicator, is unreliable on its own. There may be multiple reasons for this, but these are beyond the scope of this paper.

It should be noted that the quality indicators do not assess outcomes themselves. The original developers of the indicators acknowledged the impossibility of this: it would require parallel prospective individual patient assessments and follow-up, a process beset by ethical difficulties as well as high risk of changing observed practices (Hawthorne effect) [6, 29]. The expectation underlying SQE that is primarily focused on structure and process [25], as these indicators are, is that structure and process are drivers of outcomes: good promote good, and poor lead inevitably to poor.

While the study fulfilled its primary goal of demonstrating that the SQE methodology is applicable and practical in primary care, understandable to HCPs and patients without being unduly time consuming, we note a limitation and caveat in regard to this: only 53% of invited primary-care clinics agreed to participate. This was a consequence of the very reasonable reluctance of GPs to engage in research in an area not of special interest to them, but it resulted in a potential selection bias. This ought not to restrict the generalisability of the finding: while some practices might be less willing than others to accept the intrusion of SQE, this will be feasible methodology in all those that do. The study was also limited in its inclusion of only four countries, each represented to different extents (from four practices in Germany and Turkey to 15 in Portugal, with varying numbers of participating staff and patients), but, within Europe, these are diverse countries. Despite that it was not the study’s primary purpose, it uncovered opportunities for improvement in the management of headache patients in primary care. The quality indicators, as well as identifying-quality failings, can guide improvements.

## Conclusion

This study, the first evaluating headache service quality indicators in primary care in Europe, confirmed the indicators to be practical and fit for purpose, able to identify areas for improvement in pursuit of care quality. While quality criteria must be deployed at all levels within health-care-systems (and this study has confirmed that they can be), primary care is the setting of greatest importance since it is where management of the majority of headache-patients can and should be based [1, 4, 13]. This study, in the context of the collaborative LTB/EHF SQE project of which it is part, is a step towards bringing headache service quality centre-stage.

The next step is benchmarking studies, defining reasonable expectations of quality, and what constitutes “good” in this context, in each setting.

## Supplementary Information

**Additional file 1.** Doctors´ questionnaire.

**Additional file 2.** Service managers´ questionnaire.

**Additional file 3.** Other health-care providers´ questionnaire.

**Additional file 4.** Secretaries’ / administrators’ questionnaire.

**Additional file 5.** Patients´ questionnaire.

**Additional file 6.** Non-expert records review.

**Additional file 7.** Quality indicators.

## Data Availability

The national data sets generated by this study are not publicly accessible but are available for legitimate purposes upon request from the respective authors.

## Abbreviations

EHF: European Headache Federation
GP: General practitioner
HALT: Headache-Attributed Lost Time (index}
HCP: Health-care provider
HURT: Headache Under-Response to Treatment (questionnaire)
ICD: International Statistical Classification of Diseases and Related Health Problems
ICHD: International Classification of Headache Disorders
LTB: *Lifting The Burden*
MOH: Medication-overuse headache
SD: Standard deviation
VAS: Visual Analogue Scale
WHO: World Health Organization
WHOQoL: WHO quality of life (questionnaire)
