# Supplementary material for: Headache service quality evaluation: implementation of quality indicators in primary care in Europe
Source: J Headache Pain. 2021 Apr 28;22(1):33. doi: 10.1186/s10194-021-01236-4 (PMC8080333; doi:10.1186/s10194-021-01236-4)
Supplement: Supplementary file 5 — Additional file 5. Patients´ questionnaire. [file 10194_2021_1236_MOESM5_ESM.pdf]


Lifting The Burden
in Official Relations with the World Health Organization
The Global Campaign against Headache

UNIVERSITY OF OXFORD

Add logo of local collaborator

Research Project: Evaluation of headache service quality

[list the local collaborator(s)]

Patients' questionnaire

Under the auspices of the Global Campaign against Headache, headache centres in Europe and the United States, and the University of Oxford in the UK, are collaborating to define and measure *quality* of headache care. This is a step towards making improvements in headache care at local, national and international levels.

A definition of quality has been agreed, along with a set of measures, and these now need to be tested. A key part of testing is to ask people who have recently visited one of the collaborating centres (ie, patients) for their views and impressions of the headache service they have received.

Please help by answering this short questionnaire. Please put a tick in the appropriate box for each question. Your name will not be recorded, and your replies will not influence your treatment in any way.

Once you have completed the questionnaire, please return it to [local collaborator].

Thank you for your time.

First please indicate the date of completion: ____/____/20___

Health-care provider: please enter headache diagnosis(es) below:

	Office use only

First, about you

1. Are you…

O male?
O female?

2. How old are you?

Age __________ years

3. For how long have you been troubled by headache?

______________ days / weeks / months / years (circle one)

Now about the service that you have received

4. How much time did the doctor spend with you (including everything: enquiry, examination and conversation)? B2a

Approximate time _______________ minutes

5. Was this amount of time … B2b

O too little?
O about right?
O too long?

6. Did the doctor provide you with information about your headache and possible treatment options? D1

O yes
O no (go to 9)

7. Was the information you received understandable?

O yes
O no

8. Was the amount of information…

O too little?
O about right?
O too much?

**9. Were you reassured by the doctor?**<br>O yes<br>O no	**D2**
**10. Were you satisfied with the cleanliness and comfort of the headache clinic?**<br>O yes<br>O no	**E1**
**11. Did you feel welcomed in the doctor's surgery?**<br>O yes<br>O no	**E2**
**12. How long were you kept waiting to see the doctor?**<br>Approximate time ______________ minutes<br>**13. Was this length of time …**<br>O much too long?<br>O too long?<br>O reasonable?	**E3**
**14. Overall, what do you think about the treatment you received for your headaches?**<br>O very good<br>O good<br>O adequate<br>O poor<br>O very poor	**F1**

If you would like to add further comments, please do so here or use the back.

Thank you for completing this questionnaire. Please return it to [add details of local collaborator].
