# Supplementary material for: Headache service quality evaluation: implementation of quality indicators in primary care in Europe
Source: J Headache Pain. 2021 Apr 28;22(1):33. doi: 10.1186/s10194-021-01236-4 (PMC8080333; doi:10.1186/s10194-021-01236-4)
Supplement: Supplementary file 6 — Additional file 6. Non-expert records review. [file 10194_2021_1236_MOESM6_ESM.pdf]

Lifting The Burden

In official relations with
the World Health Organization

The Global Campaign against Headache

Application of Quality Indicators for Headache Care Services

Non-expert records review

	Indicator	Measure	Application	Evaluator
**Domain A. Accurate diagnosis is essential for optimal headache care**				
**A1**	Patients are asked about the temporal profile of their headaches	a) Duration of presenting complaint is recorded in patient's record (yes/no)<br><br>b) Frequency or days/month of symptoms is recorded in patient's record (yes/no)	a) Review of relevant fields in records of retrospective (random or consecutive) sample of patients (n=50)<br><br>b) Review of relevant fields in records of retrospective (random or consecutive) sample of patients (n=50)	a) % yes exceeds target<br><br>b) % yes exceeds target

**A2**	Diagnosis is according to current ICHD criteria	a) Diagnosis is recorded in patient's record (yes/no)<br><br>b) Diagnostic record uses ICHD terminology (yes/no)	a) Review of relevant fields in records of retrospective (random or consecutive) sample of patients (n=50)<br><br>b) Review of relevant fields in records of retrospective (random or consecutive) sample of patients (n=50)	a) % yes exceeds target<br><br>b) % yes exceeds target
**A3**	A working diagnosis is made at the first visit	Working diagnosis at first visit is recorded in patient's record (yes/no)	Review of relevant fields in records of retrospective (random or consecutive) sample of patients (n=50)	% yes exceeds target
**A4**	A definitive diagnosis is made at first or subsequent visit	Definitive diagnosis is recorded in patient's record or, if not, an appointment for review has been given (yes/no)	Review of relevant fields in records of retrospective (random or consecutive) sample of patients (n=50)	% yes exceeds target
**Domain B. Individualized management is essential for optimal headache care**				
**B4**	Treatment plans include psychological approaches to therapy when appropriate	c) Utilization of access route to psychological therapies is recorded in service records	c) Review of service records over preceding 6 months	c) Utilization rate is within predictions for setting (benchmarking)
