# Supplementary material for: Headache service quality evaluation: implementation of quality indicators in primary care in Europe
Source: J Headache Pain. 2021 Apr 28;22(1):33. doi: 10.1186/s10194-021-01236-4 (PMC8080333; doi:10.1186/s10194-021-01236-4)
Supplement: Supplementary file 7 — Additional file 7. Quality indicators. [file 10194_2021_1236_MOESM7_ESM.pdf]

# *Lifting The Burden*

In official relations with
the World Health Organization

**The Global Campaign against Headache**

# Application of
# Quality Indicators for Headache Care Services

## Notes to the following:

1. “Expert” means a physician headache specialist. According to requirement, the expert may be either from within the service (quality assurance) or external (independent audit).

2. “Target” means a quality standard set by the service itself.

3. “Benchmark” means an external quality standard set according to location and setting. (Benchmarks will be developed empirically as data become available from multiple services.)

4. The patients’ exit questionnaire may, according to setting, be:
   a) given to patients on exit from each consultation, to be filled and returned before leaving;
   b) given to patients on exit from each consultation, with stamped addressed envelope, to be filled and returned later;
   c) mailed to patients after each consultation, with stamped addressed envelope, to be filled and returned later.

| | Indicator | Measure | Application | Evaluator |
|---|---|---|---|---|
| **Domain A. Accurate diagnosis is essential for optimal headache care** | | | | |
| **A1** | Patients are asked about the temporal profile of their headaches | a) Duration of presenting complaint is recorded in patient's record (yes/no)<br><br>b) Frequency or days/month of symptoms is recorded in patient's record (yes/no) | a) Review of relevant fields in records of retrospective (random or consecutive) sample of patients (n=50)<br><br>b) Review of relevant fields in records of retrospective (random or consecutive) sample of patients (n=50) | a) % yes exceeds target<br><br>b) % yes exceeds target |
| **A2** | Diagnosis is according to current ICHD criteria | a) Diagnosis is recorded in patient's record (yes/no)<br><br>b) Diagnostic record uses ICHD terminology (yes/no)<br><br>c) Diagnosis is correct according to ICHD criteria | a) Review of relevant fields in records of retrospective (random or consecutive) sample of patients (n=50)<br><br>b) Review of relevant fields in records of retrospective (random or consecutive) sample of patients (n=50)<br><br>c) Expert review of diagnostic fields in records of retrospective (random or consecutive) sample of patients (n=50) | a) % yes exceeds target<br><br>b) % yes exceeds target<br><br>c) % exceeds target in which diagnostic information is recorded and supports diagnosis |
| **A3** | A working diagnosis is made at the first visit | Working diagnosis at first visit is recorded in patient's record (yes/no) | Review of relevant fields in records of retrospective (random or consecutive) sample of patients (n=50) | % yes exceeds target |

| | | | | |
|---|---|---|---|---|
| **A4** | A definitive diagnosis is made at first or subsequent visit | Definitive diagnosis is recorded in patient's record or, if not, an appointment for review has been given (yes/no) | Review of relevant fields in records of retrospective (random or consecutive) sample of patients (n=50) | % yes exceeds target |
| **A5** | Diagnosis is reviewed during later follow-up | Diagnostic review during follow-up is routinely undertaken (yes/no) | Enquiry of doctors | All assert yes |
| **A6** | Diaries are used to support or confirm diagnosis | a) The service has a diagnostic diary available (yes/no)<br>b) Doctors are aware of its availability (yes/no/not applicable) | a) Enquiry of service manager into availability<br>b) Enquiry of doctors into availability | a) Yes<br>b) All yes |
| **Domain B. Individualized management is essential for optimal headache care** | | | | |
| **B1** | Waiting-list times for appointments are related to urgency of need | a) A formal triage system exists (yes/no) to expedite appointments in cases of perceived urgency (yes/no)<br>b) The system is effective | a) Enquiry of doctors, service manager and appointments administrator<br>b) Expert review of waiting-list records of most recent cases of cluster headache (n=5) | a) Yes and yes<br>b) Waiting-list times are within target for setting |

| | | | | |
|---|---|---|---|---|
| **B2** | Sufficient time is allocated to each visit for the purpose of good management | a) Actual time per visit is recorded by patient in exit questionnaire<br>b) Satisfaction (yes/no) with actual time is recorded by patient in exit questionnaire<br>c) Health-care providers express overall satisfaction (yes/no) | a) Review of questionnaires from prospective consecutive sample of patients (n=50)<br>b) Review of questionnaires from prospective consecutive sample of patients (n=50)<br>c) Enquiry of health-care providers | a) % of cases exceeds target in which actual times exceed target minima<br>b) % yes exceeds target<br>c) All yes |
| **B3** | Treatment plans follow evidence-based guidelines, reflecting diagnosis | Prescribed or recommended drugs (names, doses and quantities) are recorded in patient's or service records | Expert review of diagnostic and drug fields in records of retrospective (random or consecutive) sample of patients (n=50) | % exceeds target in which drugs, doses and quantities are commensurate with diagnoses and in accordance with local guidelines |
| **B4** | Treatment plans include psychological approaches to therapy when appropriate | a) Access route to psychological therapies exists (yes/no)<br>b) Doctors are aware of its existence<br>c) Utilization is recorded in service records | a) Enquiry of service manager into availability<br>b) Enquiry of doctors into availability<br>c) Review of service records over preceding 6 months | a) Yes<br>b) All yes<br>c) Utilization rate is within predictions for setting (benchmarking) |

| | | | | |
|---|---|---|---|---|
| **B5** | Treatment plans reflect disability assessment | a) An instrument for disability assessment is available (yes/no)<br>b) Health-care providers are aware of its availability (yes/no/not applicable)<br>c) Disability is recorded in patient's record (yes/no) | a) Enquiry of service manager into availability<br>b) Enquiry of health-care providers into availability<br>c) Expert review of disability fields in records of retrospective (random or consecutive) sample of patients (n=50) | a) Yes<br>b) All yes<br>c) % yes exceeds target |
| **B6** | Patients are followed up to ascertain optimal outcome | a) The service permits follow-up as needed<br>b) Records are kept of follow-up<br>c) A follow-up diary and/or calendar is available (yes/no)<br>d) Health-care providers are aware of its availability (yes/no/not applicable) | a) Enquiry of service manager and health-care providers<br>b) Expert review of records of retrospective (random or consecutive) sample of patients (n=50)<br>c) Enquiry of service manager into availability<br>d) Enquiry of health-care providers into availability | a) All yes<br>b) % is zero of cases apparently left inconclusive without follow-up booked<br>c) Yes<br>d) All yes |

| | | | | |
|---|---|---|---|---|
| **Domain C. Appropriate referral pathways are essential for optimal headache care** | | | | |
| **C1** | Referral pathway is available from primary to specialist care | a) A usable pathway exists (yes/no)<br>b) Doctors and appointments administrator are aware of its existence (yes/no/not applicable) | a) Enquiry of service manager into availability<br>b) Enquiry of doctors and appointments administrator into availability | a) Yes<br>b) All yes |
| **C2** | Urgent referral pathway is available when necessary | a) A usable pathway exists (yes/no)<br>b) Doctors and appointments administrator are aware of its existence (yes/no/not applicable) | a) Enquiry of service manager into availability<br>b) Enquiry of doctors and appointments administrator into availability | a) Yes<br>b) All yes |
| **Domain D. Education of patients about their headaches and their management is essential for optimal headache care** | | | | |
| **D1** | Patients are given the information they need to understand their headache and its management | Satisfaction (yes/not yes) with information given is recorded by patient in exit questionnaire | Review of questionnaires from prospective consecutive sample of patients (n=50) | % yes exceeds target |
| **D2** | Patients are given appropriate reassurance | Satisfaction (yes/not yes) with reassurance given is recorded by patient in exit questionnaire | Review of questionnaires from prospective consecutive sample of patients (n=50) | % yes exceeds target |

| | | | | |
|---|---|---|---|---|
| **Domain E. Convenience and comfort are part of optimal headache care** | | | | |
| **E1** | The service environment is clean and comfortable | a) Satisfaction (yes/not yes) with cleanliness and comfort is recorded by patient in exit questionnaire<br>b) Health-care providers are satisfied with cleanliness and comfort (yes/no) | a) Review of questionnaires from prospective consecutive sample of patients (n=50)<br>b) Enquiry of health-care providers | a) % yes exceeds target<br>b) All yes |
| **E2** | The service is welcoming | Satisfaction (yes/not yes) with welcome is recorded by patient in exit questionnaire | Review of questionnaires from prospective consecutive sample of patients (n=50) | % yes exceeds target |
| **E3** | Waiting times in the clinic are acceptable | a) Satisfaction (yes/not yes) with waiting time is recorded by patient in exit questionnaire<br>b) Health-care providers are satisfied with waiting times (yes/no) | a) Review of questionnaires from prospective consecutive sample of patients (n=50)<br>b) Enquiry of health-care providers | a) % yes exceeds target<br>b) All yes |
| **Domain F. Achieving patient satisfaction is part of optimal headache care** | | | | |
| **F1** | Patients are satisfied with their management | Satisfaction (yes/not yes) with overall management is recorded by patient in exit questionnaire | Review of questionnaires from prospective consecutive sample of patients (n=50) | % yes exceeds target |
| **Domain G. Optimal headache care is efficient and equitable** | | | | |
| **G1** | Procedures are followed to ensure resources are not wasted | A protocol to limit wastage exists (yes/no) | Enquiry of service manager into existence | Yes |

| | | | | |
|---|---|---|---|---|
| **G2** | Patients are not over-investigated | Special investigations (MRI, CT, EEG, Doppler, evoked potentials, skull xray, neck xray) are recorded in service records | Expert review of service records, and of case mix from retrospective (random or consecutive) sample of patients (n=50) | Rates of investigations are within expectation for case mix and setting (benchmarking) |
| **G3** | Costs of the service are measured as part of a cost-effectiveness policy | A record of input costs exists (yes/no) | Enquiry of service manager into existence | Yes |
| **G4** | There is equal access to headache services for all who need it | A policy to ensure equal access exists (yes/no) | Enquiry of service manager and health-care providers into existence | All yes |
| **Domain H. Outcome assessment is essential in optimal headache care** | | | | |
| **H1** | Outcome measures are based on self-reported symptom burden (headache frequency, duration and intensity) | a) An outcome measure (HURT or similar) is available (yes/no)<br>b) Health-care providers are aware of its existence (yes/no/not applicable)<br>c) Outcomes according to this measure are recorded in patient's record (yes/no) | a) Enquiry of service manager into availability<br>b) Enquiry of health-care providers into availability<br>c) Expert review of outcome fields in records of retrospective (random or consecutive) sample of patients (n=50) | a) Yes<br>b) All yes<br>c) % yes exceeds target |

| | | | | |
|---|---|---|---|---|
| **H2** | Outcome measures are based on self-reported disability burden | a) An outcome measure (HALT or similar) is available (yes/no)<br>b) Health-care providers are aware of its existence (yes/no/not applicable)<br>c) Outcomes according to this measure are recorded in patient's record (yes/no) | a) Enquiry of service manager into availability<br>b) Enquiry of health-care providers into availability<br>c) Expert review of outcome fields in records of retrospective (random or consecutive) sample of patients (n=50) | a) Yes<br>b) All yes<br>c) % yes exceeds target |
| **H3** | Outcome measures are based on self-reported quality of life | a) An outcome measure (WHOQoL or similar) is available (yes/no)<br>b) Health-care providers are aware of its existence (yes/no/not applicable)<br>c) Outcomes according to this measure are recorded in patient's record (yes/no) | a) Enquiry of service manager into availability<br>b) Enquiry of health-care providers into availability<br>c) Expert review of outcome fields in records of retrospective (random or consecutive) sample of patients (n=50) | a) Yes<br>b) All yes<br>c) % yes exceeds target |

| Domain I. Optimal headache care is safe | | | | |
|---|---|---|---|---|
| **I1** | Patients are not over-treated<br>(over-treatment may mean excessive use of drugs likely to induce MOH, overdosage with potentially harmful drugs such as ergotamine or steroids, use of prophylactics for infrequent headache, use of prophylactics for the wrong diagnosis, or use of non-evidence-based treatments that are unlikely to be effective and may jeopardize safety) | a) Prescribed drugs (names, doses and quantities) are recorded in patient's record (yes/no/not applicable)<br>b) Drugs and doses are not inappropriate for most recent (working or definitive) diagnosis and setting (yes/no) | a) Expert review of treatment fields in records of retrospective (random or consecutive) sample of patients (n=50)<br>b) Expert review of treatment fields in records of retrospective (random or consecutive) sample of patients (n=50) | a) % no is zero<br>b1) % yes (not inappropriate) is 100<br>b2)-Rates of specific treatments prescribed are within predictions for the setting (benchmarking) |
| **I2** | Systems are in place to be aware of serious adverse events<br>(serious adverse events are those that cause death, are life-threatening, terminate or put at risk a pregnancy, or cause hospitalization, prolonged illness, disability and/or malignancy) | a) A system or protocol exists (yes/no)<br>b) Health-care providers are aware of its existence (yes/no/not applicable)<br>c) Serious adverse events (rates and types) are recorded by the service (yes/no)<br>d) Serious adverse events are recorded in patient's record (yes/no/not applicable) | a) Enquiry of service manager into existence<br>b) Enquiry of health-care providers into existence<br>c) Enquiry of service manager, and factual confirmation by Expert<br>d) Expert review of outcome fields in records of retrospective (random or consecutive) sample of patients (n=50) | a) Yes<br>b) All yes<br>c) Yes and yes<br>d) Serious adverse event rate is within predictions for setting-(benchmarking) |
